# Structure and Molecular Evolution of CDGSH Iron-Sulfur Domains

**DOI:** 10.1371/journal.pone.0024790

**Published:** 2011-09-16

**Authors:** Jinzhong Lin, Liman Zhang, Shaomei Lai, Keqiong Ye

**Affiliations:** 1 National Institute of Biological Sciences, Beijing, China; 2 Graduate Program in Chinese Academy of Medical Sciences and Peking Union Medical College, Beijing, China; Griffith University, Australia

## Abstract

The recently discovered CDGSH iron-sulfur domains (CISDs) are classified into seven major types with a wide distribution throughout the three domains of life. The type 1 protein mitoNEET has been shown to fold into a dimer with the signature CDGSH motif binding to a [2Fe-2S] cluster. However, the structures of all other types of CISDs were unknown. Here we report the crystal structures of type 3, 4, and 6 CISDs determined at 1.5 Å, 1.8 Å and 1.15 Å resolution, respectively. The type 3 and 4 CISD each contain one CDGSH motif and adopt a dimeric structure. Although similar to each other, the two structures have permutated topologies, and both are distinct from the type 1 structure. The type 6 CISD contains tandem CDGSH motifs and adopts a monomeric structure with an internal pseudo dyad symmetry. All currently known CISD structures share dual iron-sulfur binding modules and a β-sandwich for either intermolecular or intramolecular dimerization. The iron-sulfur binding module, the β-strand N-terminal to the module and a proline motif are conserved among different type structures, but the dimerization module and the interface and orientation between the two iron-sulfur binding modules are divergent. Sequence analysis further shows resemblance between CISD types 4 and 7 and between 1 and 2. Our findings suggest that all CISDs share common ancestry and diverged into three primary folds with a characteristic phylogenetic distribution: a eukaryote-specific fold adopted by types 1 and 2 proteins, a prokaryote-specific fold adopted by types 3, 4 and 7 proteins, and a tandem-motif fold adopted by types 5 and 6 proteins. Our comprehensive structural, sequential and phylogenetic analysis provides significant insight into the assembly principles and evolutionary relationship of CISDs.

## Introduction

Proteins with bound iron-sulfur clusters form an ancient and essential part of the proteome in every organism. Iron-sulfur proteins primarily transfer electrons in various biochemical processes, and they also take roles in substrate binding, iron-sulfur storage, gene regulation and enzyme activity [1]–[3]. The recently discovered CDGSH iron-sulfur domains (CISDs) are characterized by one or two highly conserved 17-residue CDGSH motifs with a consensus sequence of [ΦCXCXX(S/T)XXXPΦCDG(S/T/A)H], where Φ and X are a hydrophobic or any residue, respectively [4]–[8].

Humans have three CISD proteins, mitoNEET (aka CISD1), Miner1 (CISD2) and Miner2 (CISD3), which show an ill-defined mitochondria-related function. The first characterized CISD protein, mitoNEET, was initially identified in the mitochondria as a cross-linking target for the anti-diabetic drug pioglitazone [9]. The mitochondria isolated from mitoNEET knockout mice showed a reduced oxidative capacity [4]. MitoNEET consists of an N-terminal transmembrane helix and a C-terminal CISD and is located in the outer membrane of mitochondria with its CISD oriented toward the cytoplasm [4]. The crystal structure of mitoNEET CISD shows an intertwined homodimer with each subunit binding a [2Fe-2S] cluster [6]–[8]. The [2Fe-2S] cluster is coordinated with three cysteine residues and one histidine residue in the CDGSH motif. Such a 3-Cys and 1-His ligated [2Fe-2S] cluster has not been observed for other [2Fe-2S] proteins and has raised much interest in its unique physicochemical properties [10]–[14].

Miner1 is closely related to mitoNEET, sharing 44% overall sequence identity and a highly similar structure [15]. A mutation in the Miner1 gene is the causative agent for Wolfram Syndrome 2, a disease characterized by juvenile onset diabetes mellitus, optic atrophy, deafness and increased bleeding tendency [16]. Miner1 knockout mice showed growth retardation, a reduced life span and premature aging, and these phenotypes were associated with mitochondrial degeneration and respiration dysfunction [17]. Miner1 was initially reported to be localized in the endoplasmic reticulum [4], [16], but a recent study demonstrated that it primarily localizes in the outer mitochondrial membrane [17]. The third human CISD protein, Miner2, contains tandem CDGSH motifs, unlike mitoNEET and Miner1, which contain only a single CDGSH motif. The function of Miner2, which is also a mitochondrial protein [4], has not yet been described.

The iron-sulfur clusters in mitoNEET and Miner1 are redox active and may be involved in electron transfer [5], [6], [15]. Because of the histidine necessary for ligand binding, lowering the pH destabilizes the iron-sulfur cluster and reduces the redox potential [5], [15]. The stability of the iron-sulfur cluster of mitoNEET was shown to be increased by pioglitazone [7] and reduced by NADPH [18].

**Figure 1:**
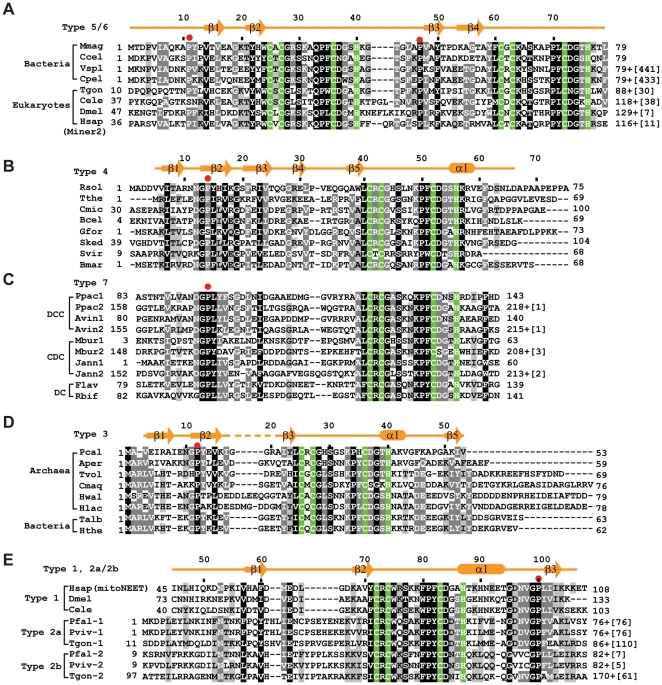
Sequence alignment of CISDs. The secondary structure elements, if available, are shown for the first protein in each alignment. The four residues coordinating the [2Fe-2S] cluster are shaded in green. Residues conserved in 100%, 80% and 60% of the displayed sequences are shaded in black, gray and light gray, respectively. The full lists of aligned CISDs are supplied as fasta files in the Supporting Information. The numbers of starting and ending residues of each sequence are labeled and the numbers of extra residues at the C-terminus are shown in brackets. Red dots indicate the lid-proline. (A) Alignment of types 5 and 6 CISDs. Mmag: *Magnetospirillum magneticum*, YP_423370 (Genebank ID); Ccel: *Clostridium cellulolyticum*, YP_002506010; Vspl: *Vibrio splendidus*, ZP_00989737; Cpel: *Candidatus Pelagibacter ubique*, YP_265568; Tgon: *Toxoplasma gondii*, XP_002369458; Cele: *Caenorhabditis elegans*, NP_497419; Dmel: *Drosophila melanogaster*, NP_610234; Hsap: *Homo sapiens*, NP_001129970. Vspl and Cpel are of type 5 and the others are of type 6. (B) Alignment of type 4 CISDs. Rsol: *Ralstonia solanacearum*, NP_521033; Tthe: *Thermus thermophilus*, YP_143292; Cmic: *Clavibacter michiganensis*, YP_001222006; Bcel: *Bacteroides cellulosilyticus*, ZP_03679950; Gfor: *Gramella forsetii*, YP_861977; Sked: *Sanguibacter keddieii*, YP_003314086; Svir: *Streptomyces viridochromogenes*, ZP_05536111; Bmar: *Blastopirellula marina*, ZP_01088744. (C) Alignment of type 7 CISDs. Different subtypes DCC, CDC and DC are indicated. The numbers 1 and 2 in the protein name indicate the first and second CISD in subtypes DCC and CDC. Ppac: *Plesiocystis pacifica*, ZP_01908188; Avin: *Allochromatium vinosum*, YP_003442661; Mbur: *Methanococcoides burtonii*, YP_566882; Jann: *Jannaschia sp. CCS1*, YP_509212; Flav: *Flavobacteriales bacterium*, ZP_02182054; Rbif: *Robiginitalea biformata*, YP_003195727. (D) Alignment of type 3 CISDs. Pcal: *Pyrobaculum calidifontis*, YP_001056297; Aper: *Aeropyrum pernix*, NP_148024; Tvol: *Thermoplasma volcanium*, NP_110689; Cmaq: *Caldivirga maquilingensis*, YP_001540894; Hwal: *Haloquadratum walsbyi*, YP_658160; Hlac: *Halorubrum lacusprofundi*, YP_002565978; Talb: *Thermocrinis albus*, YP_003472998; Hthe: *Hydrogenobacter thermophilus*, YP_003433409. (E) Alignment of type 1, 2a and 2b CISDs. Hsap, *Homo sapiens*, NP_060934; Dmel, *Drosophila melanogaster*, NP_651684; Cele: *Caenorhabditis elegans*, NP_001022387; Pfal-1 and Pfal-2: *Plasmodium falciparum 3D7*, XP_001351102 and XP_002808656; Pviv-1 and Pviv-2: *Plasmodium vivax SaI-1*, XP_001613049 and XP_001615077; Tgon-1 and Tgon-2: *Toxoplasma gondii ME49*, XP_002365250 and XP_002369458.

CISD proteins are present in the majority of eukaryotes except fungi and have a scattered distribution in prokaryotes. We have classified CISDs into seven major types based on overall sequence homology, domain composition and phylogenetic profile (Fig. 1, Table 1 and Files S1, S2, S3, S4, S5, and S6) [6]. Types 1, 2, 3 and 4 CISDs contain a single CDGSH motif and are present in eukaryotes, apicomplexa, archaea and bacteria, respectively. Type 6 CISDs possess tandem motifs separated by about 20 amino acid residues, and type 5 CISDs are a fusion of type 6 CISDs with a C-terminal glutamate synthase FMN-binding domain. One or two copies of type 7 CISDs are conjugated to a DUF1271 domain in a variable arrangement.

**Table 1:** Phylogenetic distribution of CISDs.

	No. of species belonging to a CISD type									CA-set genome^a^		
	1	2	3	4	5	6	7	O^b^	Any	CISD+	Total	%
Eukaryotes	98					56		12	122	102	324	31
Metazoan	69					44		6	72	55	120	46
Plant	12							2	14	13	32	41
Land plant	11							2	13	12	25	48
Green algae	1								1	1	7	14
Protist	5	9				12		2	22	21	41	51
Fungi									0	0	99	0
Archaea			11				10		21	18	79	23
Euryarchaeota			7				10		17	14	56	25
Crenarchaeota			4						4	4	20	20
Bacteria			7	103	32	72	43	6	255	219	1326	17
Proteobacteria			1	7	29	54	11	2	97	89	573	16
Actinobacteria				56			11		61	43	134	32
Firmicutes			1	21		2	4		27	26	299	9
Bacteroidetes			1	10	1	2	12		25	22	107	21
Cyanobacteria				1	2	6			9	9	49	18
Acidobacteria				6					6	6	11	55
Aquificae			4				2		6	6	6	100
Chlorobi				1		5			6	6	9	67
Deinococcus-Thermus				5					5	2	5	40
Planctomycetes				4				3	5	4	6	67
Fusobacteria							3		3	2	19	11
Nitrospirae						1			1	1	20	5
Spirochaetes						1			1	1	14	7
Chloroflexi				1				1	2	1	2	50
Verrucomicrobia						1			1	1	9	11

a A set of genomes that were completely sequenced or in draft assembly as listed in the NCBI genome project on December 30, 2010.

b O, orphan.

Although the highly conserved CDGSH motif will most likely bind a [2Fe-2S] cluster in all CISDs, how the iron-sulfur binding module is incorporated into structures folded by seemingly diverse sequences has previously not been understood. To date, structures are available only for the type 1 CISD proteins mitoNEET and Miner1. Preliminary crystallization results, but no structure, have been reported for a type 4 CISD [19]. To investigate the structure of different types of CISDs, we have determined high-resolution crystal structures for types 3, 4, and 6 CISDs and predicted the structures for type 2 and 7 CISDs. Our comprehensive analysis of CISD structures and sequences provides important insights into the assembly principles and evolutionary pathway of CISD-containing proteins.

## Results

### Structure determination

**Figure 2:**
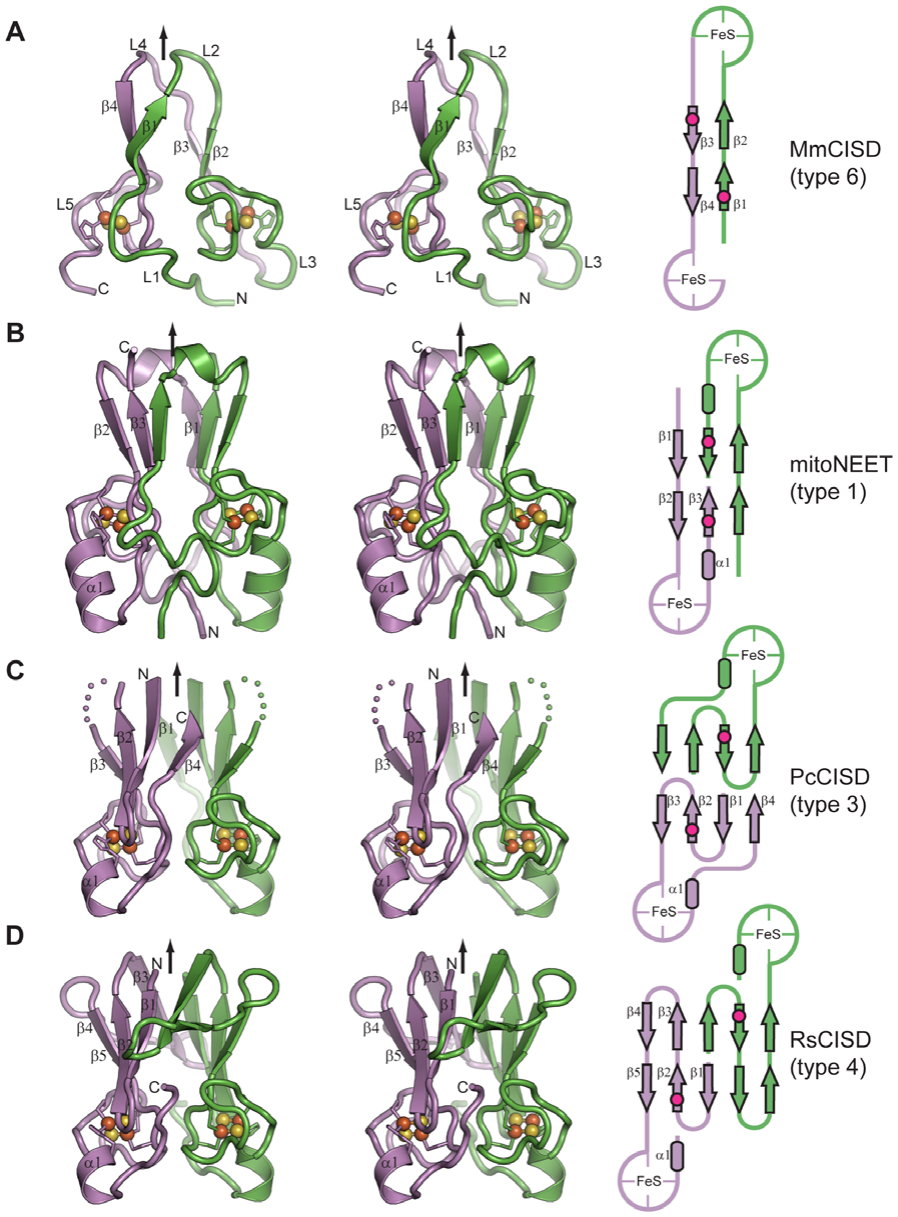
Crystal structure and topology schematic of CISDs. (A–D) Structure and topology of type 6 MmCISD (A), type 1 mitoNEET (PDB: 2QD0 [6]) (B), type 3 PcCISD (C), and type 4 RsCISD (D). Cross-eye stereo views are shown. The two subunits or two halves for the MmCISD structure are colored in green and violet. The secondary structural elements and the N- , C-terminus are indicated for one subunit. Arrows denote the dyad axis or pseudo dyad axis for MmCISD. Red dots in schematic represent the lid-proline.

We synthesized genes for a type 3 CISD (PcCISD) from *Pyrobaculum calidifontis*, which is a hyperthermophilic archaeon, a type 4 CISD (RsCISD) from *Ralstonia solanacearum*, which is a soil-born plant pathogenic bacterium, and a type 6 CISD (MmCISD) from *Magnetospirillum magneticum*, which is an aquatic alphabacterium that utilizes iron reduction to derive energy. As there is only a single CISD protein present in each of the three species' genomes, each is named CISD prefixed with the initials of the binomial name of the derived species. The three recombinant proteins display a reddish color during purification and crystallization, suggesting that they bind iron-sulfur clusters like mitoNEET and Miner1. The MmCISD structure was determined by single-wavelength anomalous dispersion (SAD) using the anomalous signal from the bound iron. The structures of RsCISD and PcCISD were solved by a combination of SAD and molecular replacement (MR) using the iron-sulfur binding module of mitoNEET as a search model. The structures of MmCISD, RsCISD and PcCISD have been refined to resolutions of 1.15 Å, 1.8 Å and 1.5 Å with an *R*_work_/*R*_free_ of 0.111/0.125, 0.208/0.254 and 0.196/0.222, respectively (Fig. 2 and Table 2). We will describe each of the three structures and derive structural themes and variation.

**Table 2:** Statistics on data collection and structure refinement.

	MmCISD	RsCISD	PcCISD
**Data collection**			
Space group	P6_5_	C2	P3_1_21
Cell dimensions			
a, b, c (Å)	63.5, 63.5, 49.1	73.2, 54.3, 43.0	49.9, 49.9, 46.1
α, β, γ (°)	90, 90, 120	90, 113.64, 90	90, 90, 120
Wavelength (Å)	1.0	1.0	1.0
X-ray source	SSRF BL17U	SSRF BL17U	SSRF BL17U
Resolution range (Å)^a^	50-1.15 (1.17-1.15)	50-1.8 (1.83-1.8)	50-1.5 (1.53-1.5)
Unique reflections	39768	13780	10747
Redundancy	9.8 (4.6)	4.3 (2.7)	11.3 (12.7)
*I*/σ	33.5 (4.9)	2.37 (9.0)	51.9 (10.4)
Completeness (%)	99.3 (97.5)	93.9 (80.9)	98.1 (100)
*R*_merge_ b	0.106 (0.494)	0.117 (0.150)	0.084 (0.487)
**Structure refinement**			
Resolution range (Å)	14.0-1.15 (1.18-1.15)	22-1.8(1.93-1.8)	20-1.5 (1.54-1.5)
No. reflections	37842	13551	10110
No. heavy atoms/hydrogen	805/614	1109	462
*R*_work_ c	0.111 (0.191)	0.208 (0.218)	0.196 (0.189)
*R*_free_ d	0.125 (0.169)	0.254 (0.278)	0.222 (0.208)
Rmsd bond length (Å)	0.005	0.012	0.010
Rmsd bond angles (°)	1.707	1.695	1.731
RAMPAGE statistics^e^			
Favoured (%)	98.8	98.3	100
Allowed (%)	1.2	1.7	0
Outlier (%)	0	0	0

a Values in parentheses are for the data in the highest resolution shell.

b *R*_merge_ = ∑|*I*_i_−*I*_m_|/∑*I*_i_, where *I*_i_ is the intensity of the measured reflection and *I*_m_ is the mean intensity of all symmetry related reflections.

c *R*_work_ = ∑|*F_o_*−*F_c_*|/∑*F_o_*, where *F_o_* and *F_c_* are the observed and calculated structure factor amplitudes.

d *R*_free_ is the same as *R*_work_, but calculated on 5% reflections not used in refinement.

e Analyzed by RAMPAGE [34].

### Type 6 MmCISD is a monomer with an internal pseudo dyad symmetry

The MmCISD structure is monomeric, with each of its two CDGSH motifs binding a [2Fe-2S] cluster (Fig. 2A). The pear-shaped structure consists of two iron-sulfur binding modules at the base and a compact β-sandwich on the top. The structure is composed of mainly loop regions (L1 though L5) interspersed by four short β-strands (β1 though β4) with a topology of L1-β1-L2-β2-L3-β3-L4-β4-L5. Strand β1 pairs with strand β4 forming a two-stranded sheet, whereas strand β2 pairs with strand β3, forming another two-stranded β-sheet, both are antiparallel. The two β-sheets pack against each other into a β-sandwich. One [2Fe-2S] cluster is coordinated by Cys25, Cys27, Cys36 and His40 in the N-terminal CDGSH motif in loop L3, whereas the other [2Fe-2S] cluster is coordinated by Cys61, Cys63, Cys72 and His76 in the C-terminal CDGSH motif in loop L5, as predicted.

**Figure 3:**
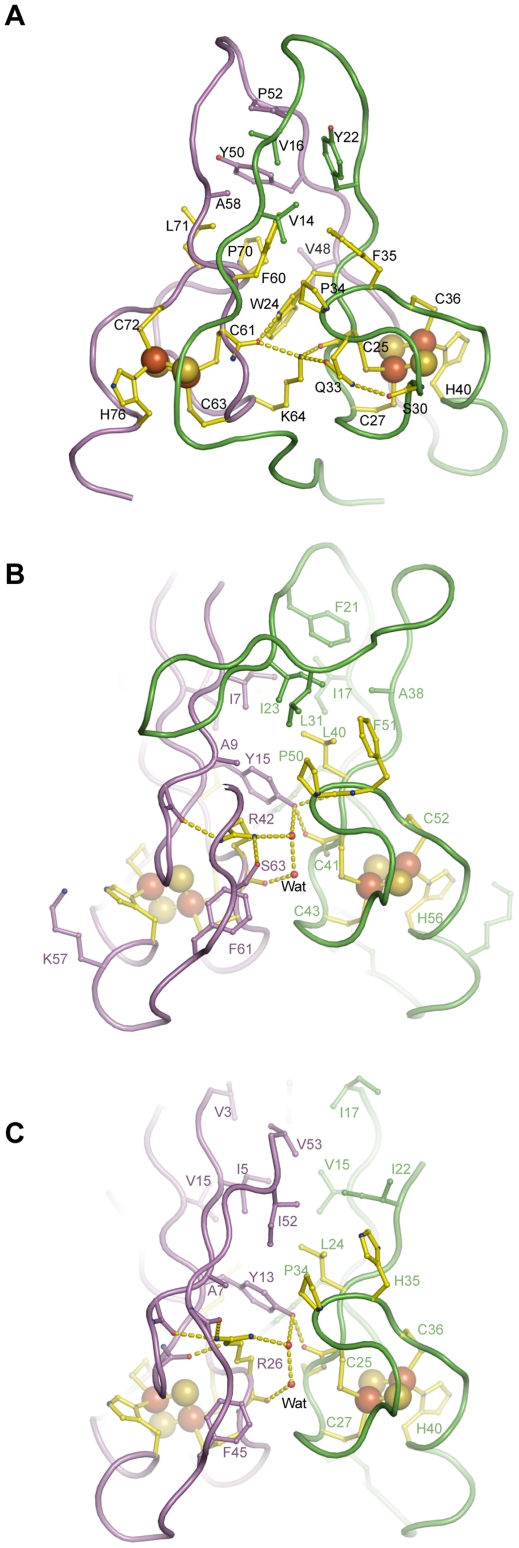
Dimer interface of CISDs. (A) The interface between the two halves of the MmCISD structure. (B) The dimer interface of the RsCISD structure. (C) The dimer interface of the PcCISD structure. Key residues are shown as sticks and balls. Oxygen atoms are red, nitrogen atoms are blue, carbon atoms in the CDGSH motif are yellow, and the remaining carbon atoms in one subunit or half are violet and those in the other subunit or half are green. Hydrogen bonds are shown as dashed lines. Only half of the interactions are displayed for the symmetric RsCISD and PcCISD dimer structures.

The monomeric structure of MmCISD displays an internal pseudo 2-fold symmetry and bears remarkable similarity to other homodimeric CISD structures. The N-terminal half (L1-β1-L2-β2-L3) is related to the C-terminal half (β3-L4-β4-L5) by a pseudo dyad axis passing through the center of the structure. The two structural halves are associated through β-strand pairing and hydrophobic and polar interactions (Fig. 3A). The major hydrophobic core is composed of Val14, Val16, Tyr22, Val48, Tyr50, Pro52, Ala58 from the β-sandwich, as well as residues Trp24, Pro34, Phe35, Phe60, Pro70 and Leu71 from the two CDGSH motifs.

The two iron-sulfur binding modules pack back-to-back with a polar interface (Fig. 3A). The interactions at the interface are asymmetric unlike those found in symmetric CISD dimer structures. The completely buried Lys64 plays a key role in bridging the two iron-sulfur binding modules. The side chain amino group of Lys64 forms three hydrogen bonds simultaneously with the backbone carbonyl oxygen of Cys61 and Cys25 and the side chain carbonyl oxygen of Gln33. The side chain amide group of Gln33 is additionally stabilized by the interaction with the carbonyl oxygen of Ser30. Residues Gln33 and Lys64 are highly conserved in the CDGSH motifs of types 5 and 6 CISDs, but not in other types of CISDs (Fig. 1), underscoring the importance of this type-specific interaction. In addition, the nitrogen atom in the indole ring of Trp24 makes a hydrogen bond with the carbonyl of Cys61, although Trp24 is often replaced by phenylalanine. Despite many polar interactions, no water molecule is found at the interface of the two tightly packed iron-sulfur binding modules.

In a search of the NCBI nonredundant database, we found type 6 CISDs in 44 eukaryotic species and 72 bacterial species, and type 5 CISDs in 32 bacterial species (Table 1). These types 5 and 6 CISDs should adopt a similar structure as MmCISD due to significant sequence homology (Fig. 1A and File S5). For instance, MmCISD and human Miner2 share 31% sequence identity and 39% sequence similarity.

Interestingly, one of the two CDGSH motifs is degenerated in 17% of types 5 and 6 CISDs (File S5). In about half of these cases, the characteristic histidine ligand is replaced by a cysteine. The resulting motif may still be able to bind a [2Fe-2S] cluster, as shown experimentally for a mitoNEET mutant [12], [20]. In the remaining cases, the iron-sulfur binding is mostly likely disrupted by substitutions (Cys to Ser, His to Gly, Ser, Gln or Asn) and whether the resultant structure with a single [2Fe-2S] cluster still folds normally is unclear.

### Type 4 RsCISD forms a homodimeric structure

Type 4 constitute a major group of CISDs present in 103 bacterial species (Table 1). RsCISD adopts an intertwined dimeric structure with the dimer interface burying 1250 Å^2^ of solvent accessible area per monomer. Residues 6–66 of RsCISD are resolved in the structure and form five consecutive β-strands (β1 though β5) followed by the iron-sulfur binding module and a short α-helix, α1 (Fig. 2D). Each subunit binds a [2Fe-2S] cluster via residues Cys41, Cys43, Cys52 and His56 in the CDGSH motif.

The structure contains a β-sandwich packed by two intermolecular five-stranded β-sheets. Each β-sheet comprises strands β1, β2 and β5 from one subunit and strand β3′ and β4′ from the other subunit with the order β5-β2-β1-β3′-β4′ (prime denotes the other subunit). The intermolecular pairing between strand β1 and β3′ is parallel, whereas all other adjacent strand pairing is antiparallel. The β-sandwich is stabilized by a large number of hydrophobic residues (Ile7, Ala9, Tyr15, Ile17, Phe21, Ile23, Leu31, Ala38, Leu40, Pro50 and Phe51), which are highly conserved in type 4 CISDs (Fig. 1B, 3B and File S4).

The two iron-sulfur binding modules are packed closer to each other in the RsCISD structure than in the mitoNEET and MmCISD structures. At the closest distance, the peptide backbone atoms of Arg42, Cys43 and Gly44 are within 3.3–3.8 Å of their counterparts. The interface between the two iron-sulfur binding modules is mainly polar, burying two arginine residues and four water molecules (Fig. 3B).

The solvent exposed residues in the RsCISD structure are mostly not conserved in type 4 CISDs with one exception, Lys57. Lys57 is located at the opening of the [2Fe-2S] cluster (Fig. 3B) and is conserved as lysine or arginine in more than 90% type 4 CISDs (Fig. 1B and File S4) and thus may be functionally important. The equivalent residue, the residue C-terminal to the histidine ligand, is not conserved in other type CISDs (Fig. 1).

### Type 3 PcCISD structure is a permutated form of type 4 RsCISD structure

Type 3 CISDs form a small group and were previously thought to exist only in archaea [6]. However, seven bacterial sequences with clear homology to the archaeal type 3 sequences should be grouped into this family (Fig. 1D, Table 1 and File S4). Like other single-motif type 1 and 4 CISDs, the 53-residue type 3 PcCISD also adopts a dimeric structure with each subunit binding a [2Fe-2S] cluster (Fig. 2C). The dimerization buries a surface area of 705 Å^2^ for each monomer. Each subunit comprises four β-strands (β1 to β4) and one α-helix (α1) arranged in the order β1-β2-β3-α1-β4. The iron-sulfur binding module is located between β3 and α1 and coordinates a [2Fe-2S] cluster with residues Cys25, Cys27, Cys36 and His40 (Fig. 3C). The β-strands from one subunit form an antiparallel four-stranded β-sheet that further associates with its counterpart from the other subunit into an intermolecular β-sandwich.

**Figure 4:**
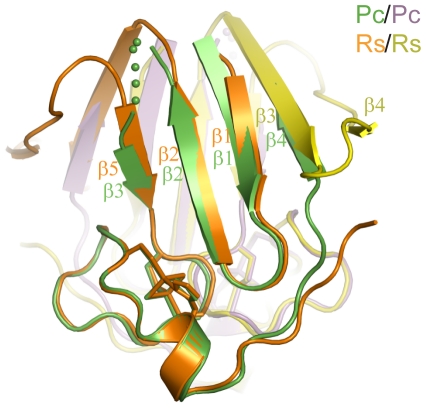
Alignment of RsCISD and PcCISD structures. Both subunits are aligned. The two subunits of PcCISD are colored in green and purple, and the two subunits of RsCISD are colored in orange and yellow.

The PcCISD structure closely resembles the RsCISD structure, but displays a permutated topology. The two structures are superimposable by both subunits with a root mean square deviation (rmsd) of 0.562 Å over 83 Cα pairs (Fig. 4). They also share similar dimer interfaces, including buried arginine residues and water molecules between the two iron-sulfur binding modules (Fig. 3B and 3C). Strands β1, β2 and β3 of PcCISD correspond to strands β1, β2 and β5 of RsCISD, respectively (Fig. 4). The β3 and β4 strands of RsCISD are replaced by a short disordered loop in the PcCISD structure; nevertheless, the position of the strand β3 of RsCISD is occupied by the C-terminal β4 strand of PcCISD coming from the opposite direction. Another difference between the two structures is that the β-sandwich of RsCISD contains the subunit-swapped β-strands β3 and β4, but there is no strand swapping in the PcCISD structure.

### Conserved structural features around iron-sulfur binding module

**Figure 5:**
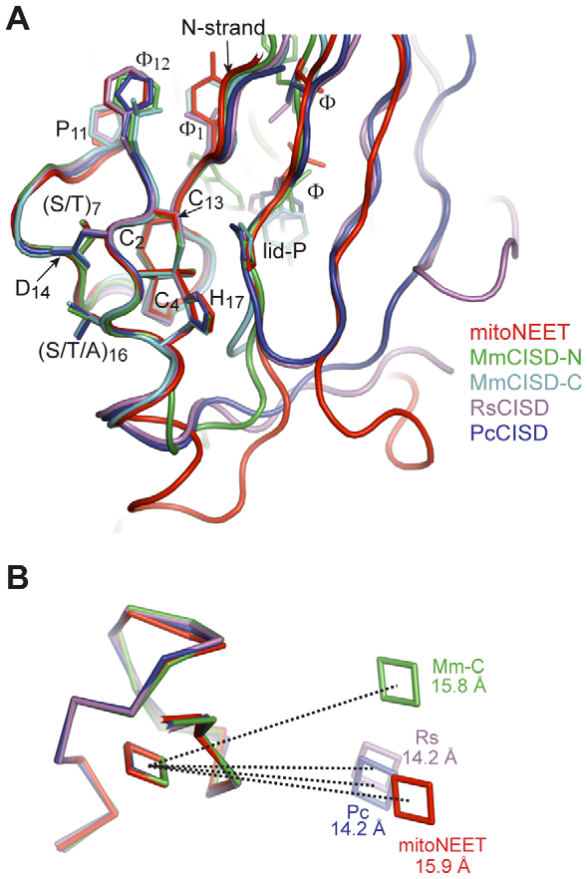
Structural conservation of CISDs. (A) Superimposition of the five nonequivalent iron-sulfur binding modules from RsCISD, MmCISD PcCISD and mitoNEET structure. Side chains are shown for the conserved residues in the CDGSH and P motif. (B) The relative positions of the two [2Fe-2S] clusters are variable in the different types of CISDs. The four structures are aligned by one CDGSH motif and the [2Fe-2S] clusters in the other iron-sulfur binding modules are shown. The centers of the two Fe-S clusters belonging to one structure are connected by dotted lines and labeled with actual distances.

The four structures of mitoNEET, MmCISD, RsCISD and PcCISD provide five nonequivalent views (two motifs from MmCISD) of how a CDGSH motif binds a [2Fe-2S] cluster. The five iron-sulfur binding modules adopt nearly identical conformations with an rmsd in the range of 0.3–0.5 Å for the 17 Cα atoms of the CDGSH motif (Fig. 5A). This is probably not surprising, given that the residues that mediate iron coordination and structure maintenance [C2, C4, (S/T)7, P11, C13, D14, G15, (S/T/A)16 and H17] are universally conserved. In addition, residues Φ1, P11 and Φ12 contribute to the hydrophobic core of the β-sandwich in all four CISD structures (Fig. 3 and 5A). This indicates that the CDGSH motif itself possesses a conserved interface for dimerization.

The structural alignment also reveals two additional conserved structural elements beyond the iron-sulfur binding module. The first element is a β-strand (called N-strand) N-terminal to the CDGSH motif that is located at the outmost part of the β-sandwich. The hydrophobic residue Φ1 of the CDGSH motif is part of the N-strand. The second element is a β-strand adjacent to the N-strand that has a consensus sequence of [GPΦXΦ]. The proline residue in the motif is located near the opening of the [2Fe-2S] cluster and lies over a bridging sulfur of the [2Fe-2S] cluster like a lid (Fig. 5A). We therefore refer to the proline as lid-proline (lid-P) and the motif as P motif. The first residue in the P motif is variable in types 5 and 6 CISDs, and the proline residue is sometimes replaced by serine in type 4 CISDs (File S4 and S5). Despite adopting a conserved structure, the P motif may occupy different positions in the primary sequence of the different types of CISDs, being N-terminal of the CDGSH motif in types 3 and 4 CISDs and C-terminal of the CDGSH motif in type 1 CISDs (Fig. 1).

### Type-specific features of CISD structure

The four CISD structures differ from other each in terms of the structure and topology of the β-sandwich dimerization module (Fig. 2). The type 6 MmCISD structure with tandem CDGSH motifs has a minimal β-sandwich packed by two two-stranded sheets and the constituent β-strands are situated N-terminal to the CDGSH motif in each half structure. The β-sandwich of type 1 mitoNEET consists of two three-stranded sheets that are assembled with β-strands from both sides of the CDGSH motif. The type 4 RsCISD structure has five-stranded sheets that are built up exclusively by the N-terminal region of the CDGSH motif. The β-sandwich of type 3 PcCISD is four-stranded, contains no swapped strand and comprises β-strands at both sides of the CDGSH motif.

In the four CISD structures, the two iron-sulfur binding modules all pack to each other in a back-to-back fashion, but their relative orientation is variable (Fig. 5B). The center-to-center distance between the two [2Fe-2S] clusters varies among 15.9 Å in mitoNEET, 15.8 Å in MmCISD and 14.2 Å in RsCISD and PcCISD. Compared to mitoNEET, when one of the iron-sulfur binding modules is aligned, the [2Fe-2S] cluster in the other module is shifted by 26 degrees in MmCISD and by 20 degrees in RsCISD and PcCISD. The different packing modes lead to certain interfacial residues in the CDGSH motif being conserved specially only in one type CISD, such as positions 3, 5, 6, and 10 in the type 1 motif, positions 5 and 10 of the N-terminal motif in types 5 and 6, position 5 in the C-terminal motif in types 5 and 6, and positions 3 and 5 in the type 4 motif (Fig. 1). The variable orientations and interactions between the two iron-sulfur binding modules suggest that the communication between dual [2Fe-2S] clusters is not essential for all CISDs.

### Structural prediction for type 7 CISD

Type 7 CISDs are characterized by one or two CDGSH motifs fused to a DUF1271 domain. We found that type 7 CISDs scatter in 10 archaeal species and 43 bacterial species (Table 1 and File S1 and S4). On the basis of domain organization, type 7 CISDs are further divided into three subtypes, in which the DUF1271 domain is followed by one CISD (subtype DC, 14 species), two CISDs (subtype DCC, 8 species) or flanked by two CISDs at either side (subtype CDC, 31 species). Irrespective of subtype and relative position to DUF1271, individual type 7 CISDs show clear sequence homology with type 4 CISDs over the full extent of the domain (Fig. 1C and File S4). This strongly suggests that type 7 CISDs fold into a type 4-like dimer. However, it remains unknown whether the two CISDs in subtypes DCC and CDC form an intramolecular dimer or form an intermolecular dimer with another molecule.

### Structural prediction for type 2a and 2b CISD

The CISD proteins from apicomplexa were previously all classified as type 2. Further analysis shows that many apicomplexa species contain up to three distinct CISD proteins. One protein contains tandem CDGSH motifs and should be classified as type 6 (such as the *T. goni* sequence in Fig. 1A). The other two are partially similar to each other and are reclassified as types 2a and 2b (Fig. 1E).

Types 2a and 2b CISDs bear significant sequence homology to type 1 CISDs within the CDGSH motif and C-terminal region (Fig. 1E, File S3). The two iron-sulfur modules are likely packed similarly in types 2a and 2b CISDs and type 1 mitoNEET, as key CDGSH motif residues mediating iron-sulfur module interaction (arginine at position 3, tryptophan at position 5 and an aromatic residue at position 10) are conserved. Moreover, the C-terminal region harbors a P motif, suggesting that types 2a and 2b CISDs, like mitoNEET, employ a C-terminal β-strand to provide the lid-proline (Fig. 2B). However, the region N-terminal to the CDGSH motif does not align well to type 1 CISDs and may adopt an alternative structure. Additionally, type 2a CISDs contain a long C-terminal extension (not shown in Fig. 1E) that might fold into an additional structure. In summary, the sequence analysis suggests that types 2a and 2b CISDs are a deviated form of type 1 CISDs.

### Phylogenetic profile of CISDs

To provide insight into the evolutionary pathway of CISDs, we analyzed the phylogenetic profile of each CISD type. Except for fungi, which is notably absent of any CISD, eukaryotes contain type 1, 2 and 6 CISDs. Type 1 CISDs appear to be universally present in metazoa and land plants (Table 1 and File S1). Some metazoa are missing a CISD gene, most likely due to incomplete sequencing of the genome. Type 1 CISDs are also found in green algae (*Chlamydomonas reinhardtii*), choanoflagellate (*Monosiga brevicollis*), diatoms (*Phaeodactylum tricornutum*, *Thalassiosira pseudonana*), brown algae (*Ectocarpus siliculosus*) and oomycetes (*Phytophthora infestans*).

Type 6 CISDs are also widely distributed in metazoa, although less frequent than type 1 CISDs, but not at all present in plants. In addition, type 6 CISDs are found in protists including apicomplexa, kinetoplastida (3 species of the genus Leishmania), ciliates (*Paramecium tetraurelia*, *Tetrahymena thermophila*), amoeba (*Dictyostelium discoideum*, *Polysphondylium pallidum*) and amoebo-flagellate (*Naegleria gruberi*).

In contrast to their extensive eukaryotic occurrence, CISDs are sporadically distributed in prokaryotes. Our search found 255 bacterial species and 21 archaeal species that contain at least one CISD gene. These prokaryotes most frequently (95.2%) have a single CISD type and 13 species have two CISD types. From a set of prokaryotes whose genomes have been completely sequenced or are in draft assembly (CA-set), 17% of bacteria and 23% of archaea are CISD positive.

Interestingly, types 4 and 5 or 6 CISDs display mutually exclusive distributions at the phylum level. Type 4 CISDs are predominantly present in Gram-positive Actinobacteria and Firmicutes, whereas type 5 and 6 CISDs are mainly found in Gram-negative Proteobacteria. Distinct patterns are also evident for smaller bacterial groups. Type 4 CISDs are preferred in Bacteroidetes (together with type 7), Acidobacteria, Deinococcus-Thermus and Planctomycetes, whereas types 5 and 6 CISDs dominate in Cyanobacteria and Chlorobi. The exclusive phylogenetic profiles of type 4 and 5 or 6 CISDs are consistent with a predominant role of vertical gene transfer in spreading these genes. Nevertheless, horizontal gene transfer (HGT), which is widespread in prokaryotes [21], may contribute to the wide and sometime spontaneous occurrences of the CISD gene in extant prokaryotes. For types 3 and 7 CISDs that are present sporadically in diverse archaeal and bacterial species with no clear phylogenetic pattern, HGT is likely the major gene spreading mechanism.

Gene loss appears to be an important determinant for the limited occurence of the CISD gene in prokaryotes. For genera in the CA-set with at least five sequenced genomes and at least one CISD-positive species, only 35±21% (mean±SD) species in the same genus are CISD-positive. As species belonging to the same genus should most likely inherit the CISD gene from a common ancestor, the absence of CISD gene in sister species is highly suggestive of gene loss.

Eukaryotes may acquire CISDs from their prokaryotic ancestor or from antecedent of mitochondria, an ancient alphaproteobactrium. The latter route is even plausible given that three human CISD proteins are all associated with mitochondrial function.

## Discussion

We have determined three structures that are representative of type 3, 4, 5, and 6 CISDs. Together with the previously determined structure of type 1 mitoNEET, the current four CISD structures provide a template to understand the assembly of ∼90% of known CISD proteins that are homologous to one of these proteins. Sequence analysis of the remaining types 2a/2b and 7 CISDs further reveals their resemblance with types 1 and 4 CISDs, respectively.

Our major conclusion is that different types of CISDs are more related to each other at the structure level than their sequences initially suggest. The four CISD structures display striking similarity in their overall appearance, with dual iron-sulfur binding modules and a β-sandwich dimerization module. It appears that the CDGSH iron-sulfur binding module must exist in a pair that could result from either dimerization of a protein containing a single CDGSH motif or folding of a single polypeptide containing tandem CDGSH motifs. The obligatory dimerization of CISD is further supported by the fact that the CDGSH motif already contains a universally conserved interface (Φ1, P11 and Φ12) for dimerization. Nevertheless, the iron-sulfur binding module requires a β-sandwich dimerization module, including a conserved N-strand and P motif, to form an integral CISD structure.

**Figure 6:**
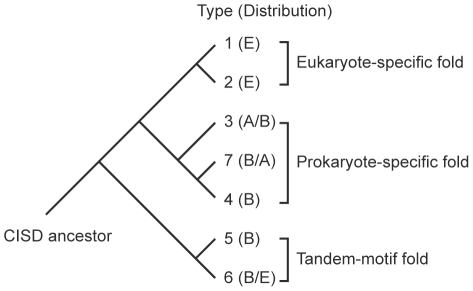
Hypothetical evolutionary relationship of the seven CISD families based on their structures and sequences. E, eukaryotes; A, Archaea; B, Bacteria.

Our analysis also shows that the seven CISD types can be architecturally reduced to three primary structural folds, which each display a distinct phylogenetic pattern (Fig. 6). Types 3, 4 and 7 CISDs adopt a prokaryote-specific fold. Among them, type 7 CISDs apparently resulted from fusion of type 4 CISDs with a DUF1271 domain, whereas type 3 CISDs are a derivative of the more predominate type 4 CISDs as revealed by their closely related structures (Fig. 4). Type 1 and 2 CISDs adopt a eukaryote-specific fold. The prokaryote- and eukaryote-specific folds are distinguished from each other by the different order of the P and CDGSH motifs in the primary sequence. The third tandem-motif fold adopted by types 5 and 6 CISDs is present across both bacteria and eukaryotes. Type 6 CISDs apparently evolved from an ancestral single-motif CISD through gene duplication and gene fusion, while type 5 CISDs resulted from fusion of type 6 CISDs with a glutamate synthase domain.

In conclusion, all CISDs appear to descend from an ancestral domain that contained a single CDGSH motif and formed a homodimer. During the course of evolution, diversification of the β-sandwich structure and gene fusion events have given rise to the three primary folds and seven major types of CISDs, while preserving the core structure of iron-sulfur binding module. The structural and evolutionary knowledge about CISDs will aid the investigation of their yet-to-be-understood function.

## Materials and Methods

### Sequence analysis

CISD proteins were searched in the NCBI nonredundant database by Pattern Hit Initiated BLAST (PHI-BLAST) using the consensus sequence of CDGSH motif as query [22]. Over 600 sequences were retrieved as of December 30, 2010, and were classified into seven major groups and an orphan group (Fig. 1, Table 1 and File S1, S2, S3, S4, S5, and S6). Some erroneous or low-quality sequences were removed. The retrieved sequences were multiply aligned with ClustalW [23]. Sequence manipulation and alignment were conducted in Jalview [24].

In the analysis of phylogeny distribution, multiple sequences of same type CISD in a species and sequences present in multiple strains were counted once. The fraction of CISD-positive genomes was calculated against a set of genomes that were completely sequenced or in draft assembly as listed in the NCBI genome project on December 30, 2010. The prokaryotic genomes in draft assembly are mostly complete, as the CISD-positive fractions calculated from the genomes in draft assembly alone are similar to those calculated from the completed genomes. However, many eukaryotic genomes in draft assembly are indeed incomplete, leading to underestimation of the fraction of CISD-positive genomes. A eukaryote that is not a metazoan, plant or fungus is regarded as a protist.

Type 7 CISDs proteins were assigned to one of the subtypes DC, CDC or DCC. The boundary of the DUF1271 domain was defined according to the structure of its homolog [25], and the individual CISD was then extracted and aligned.

### Protein expression and purification

The gene sequences encoding a type 6 CISD protein MmCISD from *Magnetospirillum magneticum* AMB-1 (access number: YP_423370), a type 4 CISD protein RsCISD (NP_521033) from *Ralstonia solanacearum* GMI1000 and a type 3 CISD protein PcCISD from *Pyrobaculum calidifontis* JCM 11548 (YP_001056297) were chemically synthesized by Generay and cloned into plasmid pET28a(+) (Novagen). Residues 2–79 of MmCISD (79 residues in total) were cloned as a fusion construct to an N-terminal 6-His tag (MGSSHHHHHH). Residues 1–66 of RsCISD (75 residues in total) were cloned as a fusion construct to an N-terminal 6-His tag and PreScission cleavage site (MGSSHHHHHHSQDLEVLFQGPH). The full-length PcCISD protein (53 residues in total) was cloned with a C-terminal 7-His tag (HHHHHHH). All proteins were expressed in *Escherichia coli* BL21(DE3) strain. The cells were cultured at 37°C to an OD_600_ of 0.8 and grew at 20°C for 16 h after protein expression was induced with 1 mM isopropyl β-D-1-thiogalactopyranoside.

For purification of His-tagged MmCISD, cells from 6 liter culture were lysed by sonication in buffer A [20 mM Tris-HCl (pH 7.9), 500 mM NaCl and 30 mM imidazole]. The clarified supernatant was loaded onto a 5-ml HisTrap column (GE Healthcare) pre-equilibrated with buffer A. The column was washed with 20 column volumes of buffer A, and the protein was eluted with a linear 30–500 mM imidazole gradient in 20 mM Tris-HCl (pH 7.9) and 500 mM NaCl. The fractions containing the target protein were concentrated and loaded onto a HiLoad 16/60 Superdex 75 column (GE Healthcare) equilibrated in 10 mM Tris-HCl (pH 7.9), 100 mM NaCl and 33 mM trisodium citrate. The protein was eluted in a broad and tailing peak. The early fractions were colorless, suggesting iron loss and protein aggregation. The late fractions (elution volume 83–90 ml) with reddish color were pooled, concentrated to 15 mg/ml, and screened immediately for crystallization.

His-tagged PcCISD was purified essentially following the same steps as MmCISD. The gel filtration with Superdex 75 was performed in buffer B [10 mM Tris-HCl (pH 7.9) and 50 mM NaCl]. Because the PcCISD protein coeluted (elution volume = 106–118 ml) with a salt peak, it was further exchanged to buffer B using a HiTrap desalting column (GE Healthcare). The protein was concentrated to 20–30 mg/ml in buffer B, flash-frozen in liquid nitrogen and stored at −80°C.

His-tagged RsCISD was purified similarly. To cleave the His-tag, the protein eluated from HisTrap column was diluted 2.5-fold with 20 mM Tris-HCl (pH 7.9) and incubated with PreScission protease overnight at 4°C. After concentrating and exchange to buffer B, the sample was passed through a HisTrap column to remove the cleaved His-tag and any uncleaved protein. The protein in the flow-though was further purified with a Superdex 75 column in buffer B. The protein was concentrated to 20–30 mg/ml, flash-frozen in liquid nitrogen and stored at −80°C.

### Crystallization

Crystallization was carried out by the hanging-drop vapor diffusion method and by mixing 1 µl of concentrated protein solution with 1 µl of reservoir solution. Crystals of MmCISD were obtained in 4.0 M sodium formate at 4°C and cryoprotected in 25% glycerol made in the reservoir solution. Crystals of RsCISD were grown in 0.5% PEG 5000, 0.9 M K/Na tartrate and 100 mM Tris-HCl (pH 7.4) at 4°C and cryoprotected in 20% ethylene glycol made in the reservoir solution. Crystals of PcCISD were grown in 1.6 M MgSO_4_ and 100 mM MES (pH 6.5) at 20°C and cryoprotected in 25% glycerol made in the reservoir solution.

### Structure determination

The initial datasets for structure determination were all recorded using a Rigaku MicroMax-007 X-ray generator equipped with an R-AXIS IV++ image plate detector at a wavelength of 1.5418 Å. These datasets have resolutions of 1.8 Å for MmCISD, 2.3 Å for RsCISD and 3.0 Å for PcCISD. Final structures were refined against data with better resolution collected at beamline BL17U of the Shanghai Synchrotron Radiation Facility (SSRF) at a wavelength of 1.0 Å. Data were processed by Denzo and Scalepack in house or HKL2000 at the synchrotron [26].

The MmCISD crystal belongs to space group P6_5_ and contains one molecule in the asymmetric unit (ASU). The structure was determined by single-wavelength anomalous dispersion (SAD) making use of the anomalous signal of iron. Heavy atom search, phase calculation and density modification were performed in SHARP [27]. The model was first built automatically by ARP/wARP [28], followed by iterations of model adjustment in Coot [29] and refinement in Refmac [30]. The final model was refined to 1.15 Å resolution in Phenix with riding hydrogen and anisotropic temperature factor for individual atoms [31]. The current model contains MmCISD residues 2–79, 9 residues from the N-terminal His-tag, two [2Fe-2S] clusters and 152 water molecules.

RsCISD crystallized in space group C2 with two molecules in the ASU. The structure was solved by combination of SAD and MR. The substructure of mitoNEET (PDB code: 2QD0) containing residues 67–88 and 99–102 and the [2Fe-2S] cluster was used as a search model for two copies with Phaser [32]. The solution was then used to locate four iron atoms. SAD phasing, MR phase combination and density modification were conducted in SHARP. The model was built in Coot and refined in Refmac. The final RsCISD structure was refined to 1.8 Å resolution with Phenix and contains residues 5–65 of each subunit, two [2Fe-2S] clusters, one polyethylene glycol molecule, one tartaric acid molecule and 105 water molecules.

The PcCISD crystal is of space group P3_1_21 and has one molecule per ASU. The structure was also solved by a combination of SAD and MR phasing. The MR search model contained residue 71–87 of the mitoNEET structure with bound [Fe-2S] cluster, where the side chains of residues that differ between mitoNEET and PcCISD were trimmed to the Cβ atom. A marginal solution with TFZ = 5.7 was found in Phaser. The anomalous map based on this solution showed two scatters, whose positions are consistent with the two iron atoms in the [2Fe-2S] cluster. The iron positions were used to calculate SAD phases in SHARP. The MR and SAD phases were combined and further improved by solvent modification, yielding an interpretable map at 3.0 Å. The model was manually built in Coot and refined in Refmac. The final model of PcCISD was refined to 1.5 Å resolution and contains residues 3–53, 6 histidine residues from the C-terminal His-tag, one [2Fe-2S] cluster and 40 water molecules. Structural figures were generated in PyMOL [33].

### Protein Data Bank accession number

The atomic coordinates and structure factors have been deposited in the Protein Data Bank under accession number 3TBO for the PcCISD structure, 3TBM for the RsCISD structure and 3TBN for the MmCISD structure.

## Supporting Information

File S1 Collection of CISD proteins with taxonomy information (Excel table).
(XLS)

File S2 Aligned sequences of type 1 CISDs in FASTA format. The file can be viewed and manipulated with Jalview [24]. Each protein name is composed of five underline-joined strings: CISD type, phylum abbreviation (or class abbreviation for proteobacteria), initial of genus name, species name and Genebank ID. Phylum abbreviations are defined as follows. Eukaryotes: Arth, Arthropoda; Baci, Bacillariophyta; Cnid, Cnidaria; Chor, Chordata; Chlp, Chlorophyta; Echi, Echinodermata; Nema, Nematoda; Phae, Phaeophyceae; Plac, Placozoa; Plat, Platyhelminthes; Stre, Streptophyta; Prot, Protist. Prokaryotes: Acid, Acidobacteria; Acti, Actinobacteria; Apro, Alphaproteobacteria; Aqui, Aquificae; Bact, Bacteroidetes; Bpro, Betaproteobacteria; Chlf, Chloroflexi; Chlo, Chlorobi; Cyan, Cyanobacteria; Dein, Deinococcus-Thermus; Dpro, Deltaproteobacteria; Epro, Epsilonproteobacteria; Firm, Firmicutes; Fuso, Fusobacteria; Gpro, Gammaproteobacteria; Nitro, Nitrospirae; Plan, Planctomycetes; Spir, Spirochaetes; Verr, Verrucomicrobia; Zpro, Zetaproteobacteria; Eury, Euryarchaeota; Cren, Crenarchaeota.
(TXT)

File S3 Aligned sequences of type 2 CISDs in FASTA format. Each protein is named as described for File S2.
(TXT)

File S4 Aligned sequences of type 3, type 4 and type7 CISDs in FASTA format. Each protein is named as described for File S2. Type 7 is further divided into subtypes CDC, DCC and DC. The first and second CISD in subtypes CDC and DCC are marked by 1 and 2, respectively.
(TXT)

File S5 Aligned sequences of type 5 and type 6 CISDs in FASTA format. Each protein is named as described for File S2. The types 5 and 6 CISDs with a degenerated CDGSH motif are labeled with prime. Eukaryotic proteins are prefixed with “e”.
(TXT)

File S6 Aligned sequences of orphan CISDs in FASTA format. Each protein is named as described for File S2.
(TXT)
